# Novel Antidiabetic Medications in Polycystic Ovary Syndrome

**DOI:** 10.15190/d.2022.4

**Published:** 2022-03-31

**Authors:** Manoj Reddy Somagutta, Molly Jain, Utkarsha Uday, Siva K. Pendyala, Ashwini Mahadevaiah, Greta Mahmutaj, Nagendrababu Jarapala, Mohamed A. Gad, Pathan Mayur Srinivas, Nayana Sasidharan, Nafisa Mustafa

**Affiliations:** ^1^Avalon University School of Medicine, Willemstad, Curacao; ^2^Saint James School of Medicine, Park Ridge, Illinois, USA; ^3^West Bengal University of Health Sciences, Kolkata, India; ^4^Jagadguru Sri Shivarathreeshwara (JSS) University, Mysore, India; ^5^The University of Medicine, Tirana, Albania; ^6^Atlantic University School of Medicine, Gross islet, St. Lucia; ^7^Saint George's School of Medicine, St. George's, Grenada; ^8^Academy of Medical Sciences, Pariyaram, Kerala, India; ^9^The National Ribat University Khartoum, Sudan

**Keywords:** Polycystic ovary syndrome, PCOS, glucagon-like peptide-1 receptor antagonists, sodium-glucose transporter-2 inhibitors, diabetes, incretin, insulin sensitivity.

## Abstract

Polycystic ovary syndrome is a very common endocrine disorder prevalent in premenopausal women. Patients with polycystic ovary syndrome present with abnormal menstruation, ovulation disorders, and hyperandrogenemia. They are often accompanied by insulin resistance, metabolic disorders, and other cardiovascular abnormalities. Also, they have comorbidities, such as dyslipidemia, obesity, diabetes type 2, non-alcoholic fatty liver disease, which all influence the treatment plan. Metformin has been defined as a treatment option in patients with polycystic ovary syndrome. However, the clinical responses to metformin are limited. Thus, the need for novel treatments with a broad range of coverage for the complications is warranted. Sodium-glucose co-transporter 2 inhibitors, glucagon-like peptide-1 receptor agonists, incretin analogs are novel drugs approved for treating type-2 diabetes. Because of their recorded benefit with weight loss, improved insulin resistance, and cardiovascular benefits in recent studies, they may help polycystic ovary syndrome women address the polycystic ovary syndrome-related risk of metabolic, reproductive, and psychological consequences. Limited literature is available on the safety and efficacy of these novel antidiabetic drugs in patients with polycystic ovary syndrome. Thus, this review is investigating the role and effectiveness of novel antidiabetic medication as an early therapeutic option in polycystic ovary syndrome.

## SUMMARY

*1. Introduction*

*2. Complications in polycystic ovary syndrome*

*3. Metformin in polycystic ovary syndrome*

*4. Sodium-glucose co-transporter 2 inhibitors in polycystic ovary syndrome*

*5. Glucagon-like peptide-1 receptor agonists in polycystic ovary syndrome*

*6. Incretin-based therapies in polycystic ovary syndrome*

*7. Conclusion*

## **1. **Introduction

Polycystic ovary syndrome (PCOS) is the most common endocrinological disorder due to hormonal dysfunction in females of reproductive age. With prevalence ranging between 5-15%, PCOS remains a multifactorial disorder that still requires extensive research in management techniques and how it affects bodily functions widely^1^. A higher prevalence of PCOS is seen in Mexican-Americans than non-Hispanic whites and African Americans^2^. The diagnosis of PCOS is usually clinical and based on the exclusion of other disorders. PCOS is diagnosed with two out of three criteria: chronic anovulation, hyperandrogenism (clinical or biological), and polycystic ovaries^3^. The hypothesis behind the etiology of PCOS, that individuals with a genetic predisposition exposed to certain environmental factors such as obesity and insulin resistance^3^. Dysregulation of androgen secretion with an over-response of 17-hydroxyprogesterone (17-OHP) to gonadotropin stimulation leads to functional ovarian hyperandrogenism (FOH)^4^. FOH-associated PCOS presents with symptoms of hyperandrogenism, oligoanovulation, and polycystic ovaries^3^. The consequences of these hormonal imbalances include insulin excess, which sensitizes the ovary to luteinizing hormone (LH), causing an intrinsic imbalance among intraovarian regulatory systems^4^. Clinical hyperandrogenism is diagnosed in adult women with hirsutism, alopecia, and acne. All these symptoms can help identify PCOS patients^1^. Furthermore, PCOS and its effect on endocrine dysregulation functions can cause significant outcomes that could be life-threatening, including cardiovascular disease, endometrial cancer, metabolic syndrome, and type-2 diabetes mellitus (T2DM)^4^. PCOS becomes a controversial and complex disorder to treat and may require the expertise of several specialists, long duration, and regular follow-ups for diagnosis and proper management.

Treatment of PCOS is also complex and requires either a single or combination of medications and therapies. Lifestyle modification with weight loss can improve anovulation due to obesity and even improve insulin sensitivity in PCOS patients^3^. Other medications, such as oral contraceptives (OCPs), clomiphene citrate, and thiazolidinediones are used to treat symptoms of hyperandrogenism and may be used in combination with anti-diabetic agents to promote an increased therapeutic effect of management may also play a role in improving infertility and reproductive function in PCOS patients by stimulating ovulation^4^. Since obesity and insulin resistance are major contributors to complications in PCOS patients, anti-diabetic agents may effectively promote insulin action, thereby promoting weight loss and improving T2DM^3^. However, due to metabolic derangements, the risk of cardiovascular diseases, liver disease, and other metabolic syndromes is high in PCOS patients. The commonly used OCPs further adds to the risk of dysglycemia and cardio-metabolic risk factors. Current guideline treatment strategies for PCOS do not effectively target these risk factors and complications, and new investigations are mandated. Studies of newer glucose-lowering agents, such as glucagon-like peptide-1 receptor antagonists (GLP-1 RAs) and sodium-glucose transporter-2 inhibitors (SGLT2i) and incretin analogs, have been promising in managing the metabolic complications of PCOS^4^. This review aims to study the effectiveness of novel antibiotic medication use in PCOS patients.

## 2. **Complications in polycystic ovary syndrome**

PCOS is a multifactorial disease that bears multiple comorbidities and long-term implications to account in the future. Some complications require a multidisciplinary approach for management and follow-up, leading to fatal consequences. Significant complications include infertility, obesity, metabolic syndrome, T2DM, cardiovascular disease, depression, nonalcoholic fatty liver disease (NAFLD), and endometrial cancer^3^.

Patients with PCOS are prone to hyperandrogenism with abnormally increased testosterone production^5^. Increased testosterone, in turn, increases the production of insulin in the body, causing hyperinsulinemia that is associated with multiple pathophysiological mechanisms disrupting the body’s metabolic pathway^4^. Hyperinsulinemia can lead to prediabetes and T2DM^3-5^. PCOS patients have a prevalence of 40% of developing impaired glucose tolerance and T2DM^4^. Moreover, insulin can increase the production of theca cells in the ovaries that produce androgens leading to hirsutism^5^. Theca cells enhance steroidogenesis and stimulate fat accumulation^5^. Furthermore, patients with PCOS have a higher prevalence of obesity than healthy individuals, although the number varies among different countries and ethnic groups^6^. Obesity is a significant risk factor for insulin resistance leading to T2DM^3-6^.

Interestingly, PCOS patients have higher upper body fat distribution, which is the area for most metabolic fat-relevant adipose tissue^5^. Visceral obesity is a leading factor behind impaired glucose tolerance and anovulation in females with PCOS^6^. An approximate prevalence of about 61% of obesity in PCOS patients across different countries and ethnic groups in comparison to healthy individuals is seen^5^. Moreover, excess central obesity can dysregulate the lipid levels contributing to hyperlipidemia with low high-density lipoprotein (HDL) levels^6^. The synergistic adverse effect of obesity and insulin resistance is remarkable to the progression of PCOS complications, such as cardiovascular disease^5^. The classic risk factors for heart disease, such as hypertension, dyslipidemia, obesity, and diabetes and the non-classic risk factors, such as C-reactive protein, homocysteine, and tumor necrosis-alpha, are increased to greater folds in patients with PCOS^7^. Hyperandrogenic PCOS patients are at higher chances of developing metabolic syndrome later in life than non-hyperandrogenic PCOS patients, which further causes the domino effect of worsened cardiometabolic profile^5^. The estimated prevalence of metabolic syndrome is 23.8%-53.3% in females with PCOS^1^. The Endocrine Society recommends that every PCOS patient be assessed for cardiovascular risk factors and global cardiovascular disease risk by regular blood pressure measurements, lipid profile screening, and glucose tolerance testing in patients with PCOS, as they are prone to developing metabolic syndrome and cardiovascular disease.

**Figure 1:**
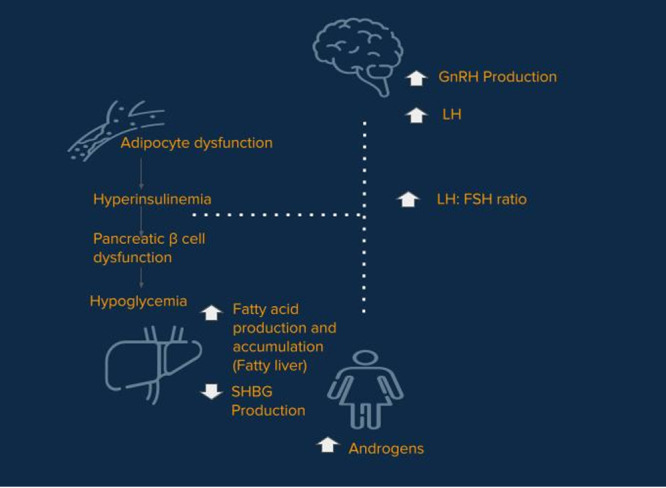
Pathophysiological mechanism of polycystic ovarian syndrome *Abbreviations*: Polycystic ovarian syndrome (PCOS); gonadotropin-releasing hormone (GnRH); sex hormone binding globulin (SHBG); luteinizing hormone (LH); follicle stimulation hormone (FSH)

Obesity in PCOS can also make patients susceptible to nonalcoholic fatty liver disease (NAFLD). PCOS patients have a three times greater risk of developing NAFLD than the average population^3^. Compilation of data from various studies demonstrates a higher prevalence of NAFLD in women with PCOS ranging from 34% to 70% compared with 14% to 34% in healthy women^8^. Inversely, women with NAFLD are more often diagnosed with PCOS^8^. NAFLD in PCOS can result from various disrupted pathological pathways surrounding hyperandrogenism, insulin resistance, and obesity^8^. Insulin resistance can lead to uncontrolled lipolysis, enhancing free fatty acids in the liver^9^. Additionally, hyperandrogenism can lead to obesity that can further cause increased fatty acid production and low sex hormone-binding globulin, further contributing to NAFLD progression as shown in Figure 1^3,8^. PCOS patients with evident risk factors for NAFLD can be screened by liver function testing and abdominal ultrasound. However, it is not required as a regular protocol since both sensitivity and specificity of NAFLD diagnosis remains low^3,8^.

## **3. **Metformin in polycystic ovary syndrome

Metformin belongs to the class of biguanide insulin sensitizers commonly used as the first line agents for T2DM for many years^10^. It improves the therapeutic effect of insulin. However, it does not affect its secretion^10^. The mechanism of action of metformin is the suppression of gluconeogenesis by the liver and the improvement of glucose uptake by the liver and skeletal muscles, thus improving insulin sensitivity^3,10^. A rat model study by Xing et al. described the effectiveness of metformin in PCOS glucose regulation^11^. Metformin improves insulin resistance by regulating the liver PI3K/AKT pathway, reducing the deposition of hepatic triglycerides, and upregulating the levels of sex hormone binding globulin (SHBG) and HNF-4α in PCOS with insulin resistance rat liver tissue^11^.

PCOS patients, particularly obese females, are inclined to hyperinsulinemia, increasing the risk of T2DM^3,4,10^. For such reasons, metformin has been used since 1994 for PCOS treatment and has effectively improved PCOS complications^10^. Various studies have shown that metformin not only helps reduce weight in PCOS patients, but also improves both endocrine and ovarian functions^12,13^. Another study by Haederi et al. reports how metformin is significant in refining endothelial function in PCOS patients^14^.

A study by Hickey et al. found that, in overweight women, metformin can have a regulatory effect on PCOS sex hormones, stimulate luteinizing hormone secretion and ovulation, and improve the menstrual cycle of patients^15^. In a systematic review by Naderpoor et al., it was evident that metformin as an adjunct with lifestyle modification is more effective in better control of obesity in PCOS patients with lower body mass index (BMI) that improves anovulation and even impaired glucose tolerance^16^. Teede et al. highlighted the comparison between combined OCPs and metformin in the management of PCOS^17^. The review further suggested that metformin alone can be an effective agent in women with BMI>25 to manage weight, hormonal and metabolic dysregulations compared to combined OCPs^17^. A recent systematic review by Guan et al. suggested that metformin can reduce body mass index, waist circumference, follicle-stimulating hormone (FSH), luteinizing hormone (LH), low-density lipoprotein (LDL), and testosterone levels in overweight women with PCOS^18^. However, not much improvement was found in LDL cholesterol levels, HDL cholesterol levels, SHBG levels, FSH levels, androstenedione levels, or triglyceride levels^18^. The review concluded metformin as the most effective intervention for PCOS in overweight women by evidencing improvement of BMI, waist circumference, and LDL cholesterol^18^. This may be due to the direct regulation effect of metformin on the production of ovarian steroids^18^.

Although metformin has been a traditional drug treatment in the class of insulin sensitizers, not many studies have confirmed its therapeutic effectiveness on very superior BMIs or reducing central adiposity, which is a key feature of metabolic syndrome^19^. In several studies, newer glucose-lowering agents, such as GLP, revealed a greater reduction of body weight, improved menstrual frequency, and improvement of hyperandrogenemia, and metabolic derangements in PCOS patients than metformin^20^. Moreover, metformin fails to add cardiovascular safety benefits and improve the lipid profile^20^. It has been recently suggested that although not yet approved, SGLT2i can provide beneficial cardiovascular and glycemic control, which are often the bigger issues in patients with PCOS^20^.

## **4. **Sodium-glucose co-transporter 2 inhibitors in polycystic ovary syndrome

Sodium-glucose co-transporter-2 receptor (SGLT-2) reabsorbs both glucose and sodium in the proximal renal tubule and is responsible for about 90% of the glucose reabsorption in the nephron. SGLT2 inhibitors are therapeutic agents to treat hyperglycemia in type 2 diabetes mellitus (T2DM) patients. These agents were also shown to be promising treatment options for patients with heart failure and kidney diseases in diabetics and even in the absence of diabetes^21^. Currently, no dedicated studies confirm the superior effects of metformin on BMI reduction or decrease in central adiposity^19^.

**Figure 2:**
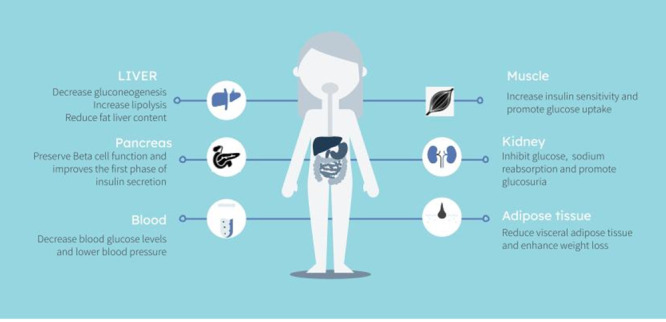
Summary of Potential benefits of SGLT-2 inhibitors

SGLT receptors inhibition can improve the disrupted metabolic status in certain PCOS patients. SGLT-2 inhibitors act on the proximal convoluted tubule of the kidney and inhibit glucose and sodium reabsorption, causing a reduction in blood glucose levels, glucosuria, and natriuresis. These medications promote glucose urine excretion leading to loss of 240-320 kcal/day, facilitating weight loss. The action of SGLT-2 inhibitors does not depend on insulin secretion, beta-cell function, or insulin resistance. They further reduce blood glucose levels by decreasing gluconeogenesis in the liver, improving insulin sensitivity andthe first phase of insulin release from the pancreatic beta-cells. It ultimately promotes glucose uptake in the muscle^19^. The potential benefits of SGLT2i are summarized in Figure 2.

In a hyperandrogenic PCOS model, empagliflozin reduced fat mass, plasma leptin, and BP. However, it failed to decrease plasma insulin, HbA1c, or albuminuria instead of its proven HbA1c lowering effects consistently shown in humans and rodent models of T1DM and T2DM^22,23^. Until now, only a few human trials are conducted to evaluate the effectiveness of SGLT2i in PCOS patients^23-26^.

Javed et al. conducted an RCT to compare the effects of empagliflozin vs. metformin on anthropometric and body composition hormonal and metabolic parameters in women with PCOS among women with PCOS. After 12 weeks of treatment with empagliflozin, they noticed a significant improvement in anthropometric parameters and body composition compared to metformin. However, no significant changes were observed in hormonal or metabolic parameters^23^. Besides reducing glucose and insulin, Tan et al. demonstrated a reduction in androstenedione and dehydroepiandrosterone sulfate concentrations, post two weeks licogliflozin treatment, a dual SGLT1/2i in a placebo-controlled RCT^24^. Cai et al. conducted a non-inferiority trial to determine the efficacy of canagliflozin vs. metformin in women with PCOS. They noticed that canagliflozin was not inferior to metformin in improving menstrual patterns, reducing body weight (BW) and total fat mass. In fact, it showed superior results in lowering uric acid and dehydroepiandrosterone sulfate (DHEAS) levels in these patients^25^.

In a recent RCT by Elkind-Hirsh et al., exenatide, when co-administered with dapagliflozin and phentermine-topiramate extended-release, exenatide alone or in combination with dapagliflozin resulted in significant improvements in mean glucose levels, improved insulin sensitivity and secretion^26^. Recently, Sinha et al. performed a meta-analysis of the above four randomized trials and reported that SGLT2i offered significant improvements in the reduction in BW, fasting plasma glucose, insulin resistance as assessed with the homeostasis model assessment-estimated insulin resistance (HOMA-insulin resistance) and DHEAS levels. However, no significant difference was observed for free androgen index, total testosterone, and SHBG^27^.

The hyperinsulinemia-induced elevation in DHEAS could indicate the risk of developing T2DM in the future^28^. A reduction in DHEAS levels, especially with increasing age, is protective against CV events^29^. Interestingly, SGLT-2, by their unique ability to reduce body weight and improve glucose uptake, would reduce hyperinsulinemia. Also, the proven ability of SGLT2i to reduce the DHEAS, delivers an intriguing thesis where reduced DHEAS causes a reduction of free testosterone, which would improve glucose utilization, forming a base for breaking down the vicious cycle of hyperinsulinemia and hyperandrogenism, the very ground of PCOS^27^. Most common adverse events reported with SGLT2 inhibitors use, include genital infections, genitourinary tract infection, vulvovaginal candidiasis, gastrointestinal symptoms, and vulvovaginitis^23-26^. This suggests SGLT2i could be an effective treatment for obese women with PCOS.

## 5**. **Glucagon-like peptide-1 receptor agonists in polycystic ovary syndrome

Glucagon-like peptide 1 (GLP-1) is a peptide hormone secreted by intestinal L cells that promote insulin secretion. It has various physiological effects, such as improving insulin resistance, inhibiting appetite and food intake, delaying gastric emptying, and reducing BW^20^. GLP-1RAs are a class of novel anti-diabetic agents that share similar effects with incretin mimetics, including glucose-dependent enhancement of insulin secretion and islet B cell proliferation^30^. GLP-1 RAs improve the insulin sensitivity in muscle and liver by directly inhibiting macrophage infiltration, thus disrupting the inflammatory pathway^20^. Animal experiments indicate that liraglutide regulates the ovarian phosphoinositide 3-kinase (PI3K)/AKT pathway to affect forkhead box protein O1 (FoxO1) phosphorylation and ovarian follicular development. GLP-1RA bind with the receptor in various regions of the hypothalamus, such as the arcuate nuclei, to inhibit appetite, increase satiety sensation, and reduce food intake. Receptor binding also delays gastric emptying and bowel movements and reduces BW through a central mechanism mediated mainly by the vagus nerve. The GLP-1RA, liraglutide reduces fat accumulation in the viscera by acting on invariant natural killer T cells, increasing the browning of white adipose tissue and enhancing fibroblast growth factor 21(FGF-21) expression in adipose tissue^31^.

Several randomized trials have been conducted to determine the effectiveness of GLP-1RAs on weight loss in obese/overweight PCOS patients as their primary outcome^32-36^. A significant weight loss has been noticed, ranging from 4.2% to 6.2% of their body weight consistently in all these studies. In a 12-week randomized study with 45 obese women with PCOS, short-term monotherapy with liraglutide was associated with significant weight loss in obese PCOS patients. Liraglutide was found to be superior to metformin and improved body composition, including a substantial visceral adipose tissue area (VAT) decrease^33^. Another randomized clinical trial evaluated the effect of liraglutide on ectopic fat in 72 obese/overweight women with PCOS. They discovered that compared with placebo, liraglutide treatment reduced BW by 5.2 kg (5.6%), VAT by 18%, liver fat content by 44%, and NAFLD prevalence by two-thirds^37^. Recently, a meta-analysis was reported to evaluate the efficacy of GLP-1RA vs. metformin for patients with PCOS. By analyzing eight RCTs, GLP-1RAs were more effective in improving insulin sensitivity reducing BMI and abdominal circumference than metformin^20^.

Several trials also evaluated the effectiveness of GLP-1RAs in reducing the risk of adverse cardiovascular outcomes by evaluating cardiovascular risk biomarkers. A study with 30 obese and women with anovulation were treated with 5 mcg bd exenatide for four weeks, then 10 mcg bd for twelve weeks. They noticed an improved serum marker of endothelial function, inflammation, and clot function, reflecting an improvement in cardiovascular risk markers in these women with PCOS^38^. Another study after six months of treatment with liraglutide (1.8 mg od) resulted in 3-4% weight loss in PCOS and a significant reduction in atherothrombosis markers, including inflammation, endothelial function, and clotting^39^.

The effects of GLP1-RAs on reproductive endpoints have been assessed in a few studies. Treatment with 1.8 mg liraglutide once daily for 26 weeks in overweight women with PCOS resulted in a 19% increase in SHBG levels along with a 19% decrease in free testosterone levels, compared with placebo, without any change in total testosterone levels^37^. Nylander et al. evaluated the role of liraglutide in improving ovarian dysfunction in PCOS patients compared to placebo. After treating 72 patients for 26 weeks, the bleeding ratio improved with liraglutide, SHBG increased by 7.4 nmol/L, free testosterone decreased by 0.005 nmol/L, an ovarian volume decreased by 1.6 ml in the liraglutide group^40^. A randomized study was realized on 28 infertile obese PCOS to evaluate the effect of short-term preconception liraglutide on fertility possibility in PCOS. The SHBG levels and pregnancy rate per embryo transfer were significantly higher when metformin was combined with low-dose liraglutide than metformin alone^41^. In another study, exenatide-treated patients exhibited significantly higher natural pregnancy rates than metformin-treated patients after twelve weeks^42^. The findings of several studies are summarized in Table 1.

**Table 1:** Main Clinical Studies in PCOS^23-51^ **Abbreviations:** N/A: Not associated; AMH, anti-Müllerian hormone; cIMT, carotid-intima media thickness; EXE, exenatide; FAI, free androgen index; HOMA-IR, homeostasis model assessment-IR; hsCRP, high-sensitivity C-reactive protein; IR, insulin resistance; IVF, in vitro fertilization; LIRA, liraglutide; MET, metformin; NAFLD, nonalcoholic fatty liver disease; NICHD, National Institute of Child Health and Human Development; OGTT, oral glucose tolerance test; PIIINP, Procollagen Type III N-Terminal Peptide; SHBG, sex hormone binding globulin; VAT, visceral adipose tissue, ROF: roflumilast.

Study	Participant characteristics	Study arms	Primary outcome	Other outcomes	Metabolic changes / Body weight loss (kg)
Javed et al (12 weeks)^23^	Women with PCOS	Empagliflozin 25 mg (n = 19) or metformin 1500 mg (n = 20)	Differences in weight, BMI, waist and hip circumference, basal metabolic rate, fat mass	----	Basal metabolic rate (empagliflozin: -1.8 ± 2.9% vs metformin: 0.1 ± 1.9%, P = 0.024) / Empagliflozin: -1.4 ± 3.2% vs metformin: 1.2 ± 2.3%; P = 0.006
Tan et al (14 days)^24^	Overweight or obese and insulin-resistant women with PCOS fulfilled the Rotterdam criteria for phenotype A or B	50 mg of oral licogliflozin or matching placebo three times a day (TID) before meals	Treatment effect on the geometric mean of three serial FT samples	Effects on sex steroids including TT, A4, DHEA, DHEAS and sex hormone binding globulin (SHBG), as well as free androgen index (FAI)	1) Concentration of FT did not change 2) Androstendione (A4) reduced by 19% 3) dehydroepiandrosteron sulphate (DHEAS) reduced by 24% 4) Hyperinsulinaemia was reduced by 70% / -----
Cai et al (12 weeks)^25^	Women aged 18 to 45 years with PCOS and IR	Randomized into 100 mg (n = 33) canagliflozin daily or 1500 to 2000 mg metformin daily (n = 35)	Homeostatic model assessment (HOMA)-IR	Changes in anthropometric measurements, menstrual frequency, Sex hormone levels, metabolic variables and body fat distribution	1)Both canagliflozin and metformin significantly improved menstrual pattern, and decreased triglyceride levels. 2)Compared with metformin, canagliflozin had significant advantages in reducing uric acid and dehydroepiandrosterone sulphate levels / Both canagliflozin and metformin significantly reduced body weight and total fat mass
Elkind-Hirsch et al (24 weeks)^26^	Nondiabetic women (n = 119; aged 18-45 years) with 30<BMI<45 and PCOS (National Institutes of Health criteria)	Randomized intoEQW (2 mg weekly); DAPA (10 mg daily), EQW/DAPA (2 mg weekly/10 mg daily), DAPA (10 mg)/MET (2000 mg XR daily), or PHEN (7.5 mg)/TPM (46 mg ER daily) treatment	Changes in body weight, total body fat	Improvement in mean blood glucose (MBG), and compute insulin sensitivity (SI) and secretion (IS) measures	1)EQW/DAPA and EQW resulted in significant improvements in MBG, SI, and IS. 2)Reductions in fasting glucose, testosterone, FAI, and BP were seen with all drugs / 1)EQW/DAPA and PHEN/TPM resulted in the most loss of weight and total body fat by DXA, and WC 2)Equivalent reductions in BMI and WC with PHEN/TPM
Rasmussen et al (28 weeks)^32^	Obese (BMI ≥30) women pre-treated with MET for min 6 months	LIRA as an add-on therapy	Weight loss	----	BMI reduction of 3.2 kg/m2, Weight loss of >5% to >10% of initial weight / −9.0 (p<0.05)
Jensterle et (12 weeks) al^33^	45 Obese premenopausal women.	MET or LIRA or ROF	Weight loss	Metabolic, Hormonal, and Menstrual frequency changes	HOMA-IR decreased in all treatment arms. FAI lessening in ROF. LIRA has a greater reduction in visceral fat and glucose at 120 min of OGTT compared to MET / MET: −0.8 ± 1.0; LIRA: −3.1 ± 3.5; ROF: −2.1 ± 2.0
Jensterle Sever et al (12 weeks) ^34^	Obese women pretreated with MET	MET, LIRA, MET + LIRA	Weight loss	1)Body composition IR 2) No significant changes in menstrual pattern	LIRA+MET had a more significant reduction in glucose after 120 min of OGTT compared to MET / MET: −1.2 ± 1.4, LIRA: −3.8 ± 3.7, MET+ LIRA: −6.5 ± 2.8
Jensterle et al (12 weeks)^35^	Obese pre-menopausal women diagnosed with PCOS.	MET vs. LIRA	Weight loss	BMI and insulin resistance	Total testosterone decreased in MET group. LH increased in LIRA / LIRA superior in BMI reduction and insulin resistance compared to MET
Jensterle et al (12 weeks)^36^	Obese (BMI ≥30) women Age ≥18 years, premenopause PCOS diagnosed by ASRM-ESHRE Rotterdam criteria	1) MET 1000 mg BID + LIRA 1.3 mg OD s.c. (n = 15) 2) LIRA 3 mg OD s.c. (n = 15)	Weight loss	Metabolic and hormonal changes	Both interventions resulted in a significant decrease of post-OGTT glucose levels. Combination therapy significantly reduced total testosterone / 1) −3.6 ± 2.5 2) −6.3 ± 3.7
Frøssing et al (26 weeks)^37^	Obese women diagnosed with PCOS	LIRA vs Placebo	Liver fat, VAT and NAFLD prevalence	Weight change OGTT, SHBG, testosterone	LIRA group had liver fat content reduction by 44%, VAT by 18%, and the decreased prevalence of NAFLD by 66%. Also, SHBG levels increased, and free testosterone decreased in the LIRA group / LIRA had significant weight loss compared to placebo
Dawson et al (16 weeks)^38^	Overweight/ obese anovulatory women with all 3 Rotterdam criteria	exenatide 5 mcg bd for 4 weeks then 10 mcg bd for 12 weeks (n=30)	Weight change	Changes in endothelial function, serum endothelial markers, inflammation, and alteration in clot structure and formation.	1)No effects on LDL-C and HDL-C. 2) No effects on glucose, insulin, and HOMA-IR. 3) Serum endothelial markers changed with a reduction in ICAM-1, p-selectin and E-selectin without an overall change in endothelial function. 4)Inflammation improved significantly 5) Significant reduction in clot function but not clot structure / Approximately 4.6 kg weight loss (p< 0.05)
Kahal et al (6 months)^39^	Obese women diagnosed with PCOS vs. healthy controls	LIRA	Weight loss	CV risk markers	In both PCOS and control groups HOMA-IR, hsCRP, endothelial adhesion markers significantly reduced / Weight was significantly reduced by 3.0 ± 4.2 and 3.8 ± 3.4 kg in the PCOS and control groups respectively
Nylander et al (26 weeks)^40^	Obese women diagnosed with PCOS	LIRA vs Placebo	Bleeding pattern which improved significantly with LIRA vs. placebo	Levels of AMH, sex hormones, and gonadotrophins, ovarian morphology	In the LIRA group: SHBG increased, free testosterone decreased, and ovarian volume decreased / —
Salamun et al (12 weeks)^41^	28 infertile obese PCOS patients	MET vs. MET+ LIRA (COMBI)	The in vitro fertilization pregnancy rate was significantly higher in the COMBI (85.7%) compared with the MET (28.6%)	Weight change	---- / Weight loss with MET group: −7.0 ± 6.0; COMBI gorup: −7.5 ± 3.9
Liu et al (12 weeks)^42^	Overweight or Obese women (BMI ≥24) diagnosed with PCOS.	MET vs. EXE	Weight loss	Metabolic parameters	Greater reduction in total fat mass % with EXE and HOMA-IR and insulin levels with EXE than with MET. HsCRP levels decreased significantly in the EXE group only. Menstrual frequency increased significantly in both groups. Rate of natural pregnancy significantly higher with EXE than MET / MET: −2.28 ± 0.55EXE: −4.29 ± 1.29
Svendsen et al (8 months)^42^	Women with PCOS vs. Healthy controls	Metformin effect on incretin secretion	Incretin hormone response did not differ between subjects with and without PCOS.	1)Lower GIP levels in obese women with PCOS compared with obese control women and compared with lean women with PCOS 2)Treatment with metformin increases the levels of both GIP and GLP-1 in women with PCOS.	The incretin hormone response did not differ between subjects with and without PCOS. Subgroup analysis showed lower GIP (area under the curve [AUC]) levels in obese women with PCOS compared with obese control women (P < .05) and compared with lean women with PCOS (P < .05). Metformin increased GIP (AUC) and GLP-1 (AUC) in lean women with PCOS (P < .05), and a similar trend was seen in the obese women (P = .07) / ---
Devin et al (1 month)^48^	Women with PCOS	Sitagliptin vs. Placebo	Sitagliptin effect on growth hormone and VAT	N/A	Sitagliptin decreased the peak glucose and visceral adiposity but did not increase growth hormone / ---
Ferjan et al (12 weeks)^49^	Obese women with PCOS and metformin intolerant BMI (36.9 ± 5.5 kg/m2).	1)Sitagliptin 100 mg QD (n = 30). 2)Lifestyle intervention (placebo, n = 30).	Glycaemic control (OGTT, HOMA-B)	---	1) Sitagliptin BMI (+37 ± 6.2–37.8 ± 5.9kg/m²). 2) Placebo BMI (+36.8 ± 4.9–38 ± 5kg/m²) / ---
Frederich et al (18-48 months)^51^	Interquartile age range: 47-61 years 51% females, 73% white, 52% hypertensive, 44% hypercholesterolemic, 39% smoking history, 20% with first-degree family member with premature coronary heart disease, 2% with prior CV disease	4607 randomized and treated patients (n = 3356 treated with saxagliptin [2.5-100 mg/d]; n = 1251, comparator [n = 656, placebo; n = 328, metformin; n = 267, uptitrated glyburide])	Changes in CV death/MI/stroke	Change from baseline glycated hemoglobin (HbA1c) at week 24	No increased risk of CV death/MI/stroke was observed in patients randomly assigned saxagliptin / ---

However, nausea and headache incidence rates were higher with GLP-1RAs than with metformin. However, they were not significant^20^. In addition, with GLP-1RAs being injectable, it may be challenging for the patients to add injectable drugs to their daily or weekly routine^31^. However, GLP-1RAs might be a good choice for obese patients with PCOS, especially those with insulin resistance.

## **6. **Incretin-based therapies in polycystic ovary syndrome

Incretins (glucose-dependent insulinotropic peptide (GIP) and GLP-1) are gut hormones that are secreted from enteroendocrine cells, which increase insulin secretion from the pancreas in response to ingested food^43^. Increased GIP and lower GLP-1 concentrations have been reported after an oral glucose tolerance test (OGTT) in women with PCOS^44^. Endogenous GLP-1 is characterized by a relatively short half-life compared to GIP, since it is degraded by the proteolytic enzyme dipeptidyl peptidase-4 (DPP-4)^45^. The DPP-4 inhibitors are considered a class of anti-diabetes medications administered orally that improve glycemic control by promoting an increase in the endogenous physiological levels of GIP and GLP-1^46^. Incretins, primarily GLP-1RAs, can overcome metabolic derangements of PCOS and add cardiovascular benefits^47^.

Svendsen et al. noticed a trend towards a slightly increased incretin hormone response in lean women with PCOS compared with lean control women, partly following Vrbikova et al^43,44^. A double-blind crossover study tested the effect of sitagliptin on blood glucose levels and VAT in women with PCOS and demonstrated a reduction in the maximal glucose response to the oral glucose tolerance and VAT^48^. A randomized pilot study by Ferjan et al. examined sitagliptin as a potential treatment option in metformin intolerant PCOS patients and reported that sitagliptin improved β-cell function and insulin sensitivity^49^. These are commonly administered in subcutaneous form, which can be a limitation by many patients. On the other hand, the evidence of oral incretin therapy on cardiovascular protection is lacking^50^. Hypersensitivity reactions, including angioedema, anaphylactic, and dermatological reactions, have been reported with saxagliptin therapy, though rare^51^.

## 7. Conclusion

Polycystic ovary syndrome is a common endocrinopathy affecting women of reproductive age. A wide range of symptoms and degrees of severity are observed, giving the syndrome numerous phenotypes. The health burden associated with PCOS has driven the need for novel therapeutic strategies to prevent complications, especially dysglycemia, cardiometabolic risks, and fertility outcomes. However, recent studies with SGLT-2i have shown promising improvements in anthropometric parameters and body composition, and more significant cardiovascular and antihyperglycemic effects in patients with PCOS. Also, GLP-1RAs, unique for weight loss treatment, make up an ideal pre-treatment for overweight/obese females who carry higher risks during controlled ovarian stimulation and pregnancy. SGLT-2 inhibitors and GLP-1RAs have good therapeutic benefits in women with PCOS improving metabolic irregularities. Since no single intervention can potentially treat the full spectrum of metabolic disorders in PCOS, lifestyle intervention combined with metformin, GLP-1RA, SGLT-2i, and bariatric surgery alone or in combination may have better outcomes. Long-term, high-quality research is further needed to look into new treatments to evaluate specific outcomes based on PCOS phenotypes.

## KEY POINTS

**◊** *Polycystic ovary syndrome (PCOS) is a multifactorial disorder many time complicated with dyslipidemia, obesity, diabetes type 2, non-alcoholic fatty liver disease, metabolic syndrome, cardiovascular disease, endometrial cancer and other manifestations.*

**◊** *Obesity leading to insulin resistance is the root cause behind disruption of pathophysiology in PCOS patients.*

**◊** *Although metformin has traditionally been used for treatment of insulin resistance in PCOS patients, its low therapeutic effectiveness as suggested by various studies brings in the novel role of other insulin sensitizers into play.*

**◊** *Sodium-glucose co-transporter 2 inhibitors, glucagon-like peptide-1 receptor agonists, incretin analogs are novel drugs approved for treating type-2 diabetes. Their role in promoting weight loss, cardio-metabolic protectiveness and improving insulin sensitization makes them quite eligible treatments for PCOS patients.*

**◊** *Further research is required to uncover new treatments for specific complications based on PCOS phenotypes.*
